# Role of joint interactions in upper limb joint movements: a disability simulation study using wearable inertial sensors for 3D motion capture

**DOI:** 10.1186/s12984-024-01480-0

**Published:** 2024-11-05

**Authors:** Nishtha Bhagat, Preeti Raghavan, Vikram Kapila

**Affiliations:** 1 Mechanical and Aerospace Engineering Department, NYU Tandon School of Engineering, Brooklyn, NY 11201 USA; 2 Physical Medicine and Rehabilitation and Neurology Departments, Johns Hopkins University School of Medicine, Baltimore, MD 21287 USA

**Keywords:** Range of motion, Upper limb, Joint movement, Restriction condition, Disability simulation, Joint interactions

## Abstract

**Background:**

Restriction of movement at a joint due to disease or dysfunction can alter the range of motion (ROM) at other joints due to joint interactions. In this paper, we quantify the extent to which joint restrictions impact upper limb joint movements by conducting a disability simulation study that used wearable inertial sensors for three-dimensional (3D) motion capture.

**Methods:**

We employed the Wearable Inertial Sensors for Exergames (WISE) system for assessing the ROM at the shoulder (flexion–extension, abduction–adduction, and internal–external rotation), elbow (flexion–extension), and forearm (pronation-supination). We recruited 20 healthy individuals to first perform instructed shoulder, elbow, and forearm movements without any external restrictions, and then perform the same movements with restriction braces placed to limit movement at the shoulder, elbow, and forearm, separately, to simulate disability. To quantify the extent to which a restriction at a non-instructed joint affected movement at an instructed joint, we computed average percentage reduction in ROM in the restricted *versus* unrestricted conditions. Moreover, we performed analysis of variance and post hoc Tukey tests (*q* statistic) to determine the statistical significance (*p* < 0.05 denoted using ^*^) of the differences in ROM of an instructed joint in the unrestricted *versus* restricted conditions.

**Results:**

Restricting movement at the shoulder led to a large reduction in the average ROM for elbow flexion–extension (21.93%, *q* = 9.34^*^) and restricting elbow movement significantly reduced the average ROM for shoulder flexion–extension (17.77%, *q* = 8.05^*^), shoulder abduction–adduction (19.80%, *q* = 7.60^*^), and forearm pronation-supination (14.04%, *q* = 4.96^*^). Finally, restricting the forearm significantly reduced the average ROM for shoulder internal–external rotation (16.71%, *q* = 3.81^*^) and elbow flexion–extension (10.01%, *q* = 4.27^*^).

**Conclusions:**

Joint interactions across non-instructed joints can reduce the ROM of instructed movements. Assessment of ROM in the real-world using 3D motion capture, for example using the WISE system, can aid in understanding movement limitations, informing interventions, and monitoring progress with rehabilitation.

## Introduction

Many neurological conditions such as a stroke, which is a leading cause of disability [1], can result in upper limb impairments that include reduced range of motion (ROM) [2, 3] and loss of fractionated movements [4] in joints. Degraded movements at the shoulder, elbow, and forearm following a stroke [5] may lead to disuse of the affected limb (learned non-use) [6] during activities of daily living (ADLs). Moreover, attempted movement at an affected joint may lead to compensatory movements at other joints, which may become habitual leading to learned bad-use and further exacerbate the movement dysfunction [6].

Disability simulation [7] has been used as a strategy to investigate the effect of impairment on joint movements using a brace or splint [8–10]. Restricting elbow joint motion with the use of a splint increased compensatory motion at the shoulder and reduced movement at the forearm during three feeding activities involving the elbow joint [8]. Similarly, restricting wrist motion with a splint reduced the ROM for the wrist degrees of freedom (DOFs) and introduced compensatory movements at the shoulder, elbow, and trunk [10]. Restricting the motion of elbow, forearm, wrist, or fingers using braces was also shown to impact overall hand function [9]. Thus, disability simulation with the use of externally imposed joint restrictions can reveal the influence of restriction at a joint on the function of other joints.

A joint is expected to move less when a restriction is placed on it. However, movements that seemingly occur at a single primary or “instructed” joint, rarely occur at only that joint since interaction forces from movement at other joints and muscles can also influence the movement at the instructed joint [11]. The joint interaction torques have been shown to be altered in individuals with movement dysfunction [12–15]. These joint interactions can affect the ROM of upper limb joint movements, exacerbating the movement dysfunction, and must be considered in the treatment. Understanding how “non-instructed” joints affect the movement at an instructed joint may provide information about compensatory strategies used by individuals with joint impairments and it may guide re-training strategies to restore normal movement.

Various motion capture (MOCAP) systems are available to assess ROM for diverse applications [16]. For example, a 10-camera VICON system [17] and an inertial measurement-based MOCAP system [18] have been used to assess ROM at upper limb joints. These MOCAP systems are expensive and not readily available in clinical settings for user-friendly and quick assessments due to their reliance on equipment available only in controlled settings and the extensive offline data analyses required [16]. Alternatively, goniometers [19] and inclinometers [20] are relatively inexpensive clinical tools that are widely available but have limited inter-observer agreement [20, 21], and cannot capture movements at multiple joints and planes simultaneously [17, 22]. Video-based, marker-less MOCAP systems, like the Kinect, can capture motion in three dimensions (3D) [16]. However, the resolution of such systems for horizontal-plane body movements, such as forearm pronation-supination and shoulder internal–external rotation, which are critical for many ADLs [23], has been reported to be inadequate [22, 24].

To overcome the above limitations of traditional MOCAP systems, the Wearable Inertial Sensors for Exergames (WISE) system was developed and validated for user-friendly capture of 3D movements at the shoulder, elbow, and forearm [22, 24]. As seen from [25], BNO055 inertial sensor used in the WISE system provides better static and dynamic angular measurement stability compared to MPU9150 and X-NEUCLEO inertial sensors. Prior research [26, 27] has used inertial sensor-based commercial MOCAP systems from Noraxon and Xsens. Similar to the WISE system, these commercial MOCAP systems utilize magnetometer, accelerometer, and gyroscope units; employ a Velcro strap to mount each sensor; and require wearing one sensor module each on the forearm and upper arm. Even as the commercial MOCAP systems offer better sampling rate, battery life, and measurement accuracy, for this laboratory-based disability simulation study, the WISE system was deemed better suited for the following reasons. First, the cost of materials, supplies, and software tools required to develop the WISE system is relatively low. Second, the WISE system permits the experimenter to flexibly utilize BNO055’s built-in sensor fusion and operating modes to acquire absolute orientation in quaternion or Euler angle form. Third, the WISE system offers ease of hardware/software problem-solving due to in-house design and development experience. Fourth, the exergame platform of the WISE system offers user-friendly feedback, incorporating a realistic animation of the user, to help them understand the difference between their performance *versus* that of a virtual instructor. In contrast, the aforementioned commercial systems either utilize a human skeleton model or do not feature a virtual instructor.

In contrast to the traditional MOCAP systems, the advantages of the WISE system include: the ability to simultaneously measure 3D movements at multiple joints (and limbs) in the sagittal, frontal, and horizontal planes, and its ease of use for a clinical or home setting [22]. These features can be helpful to apply the information obtained from disability simulation studies to the neurologic population in real-world settings. Hence, we used the WISE system to capture 3D ROM at multiple upper limb joints and simulated the effect of joint impairment with the use of restriction braces at the shoulder, elbow, and forearm to quantify the effect of a restriction placed at a non-instructed joint on the movement of an instructed joint. We hypothesized that restrictions at non-instructed joints would contribute significantly to the reduction in movement at the instructed joint by altering joint interaction forces.

## Materials and methods

### Participants

Twenty right hand dominant adults (*n* = 20), between ages 23–27 years (50% female), provided written consent to participate in the study in compliance with New York University Institutional Review Board procedures (IRB-FY2020-4198). The participants were healthy with no history of movement difficulty.

### Apparatus

**Fig. 1:**
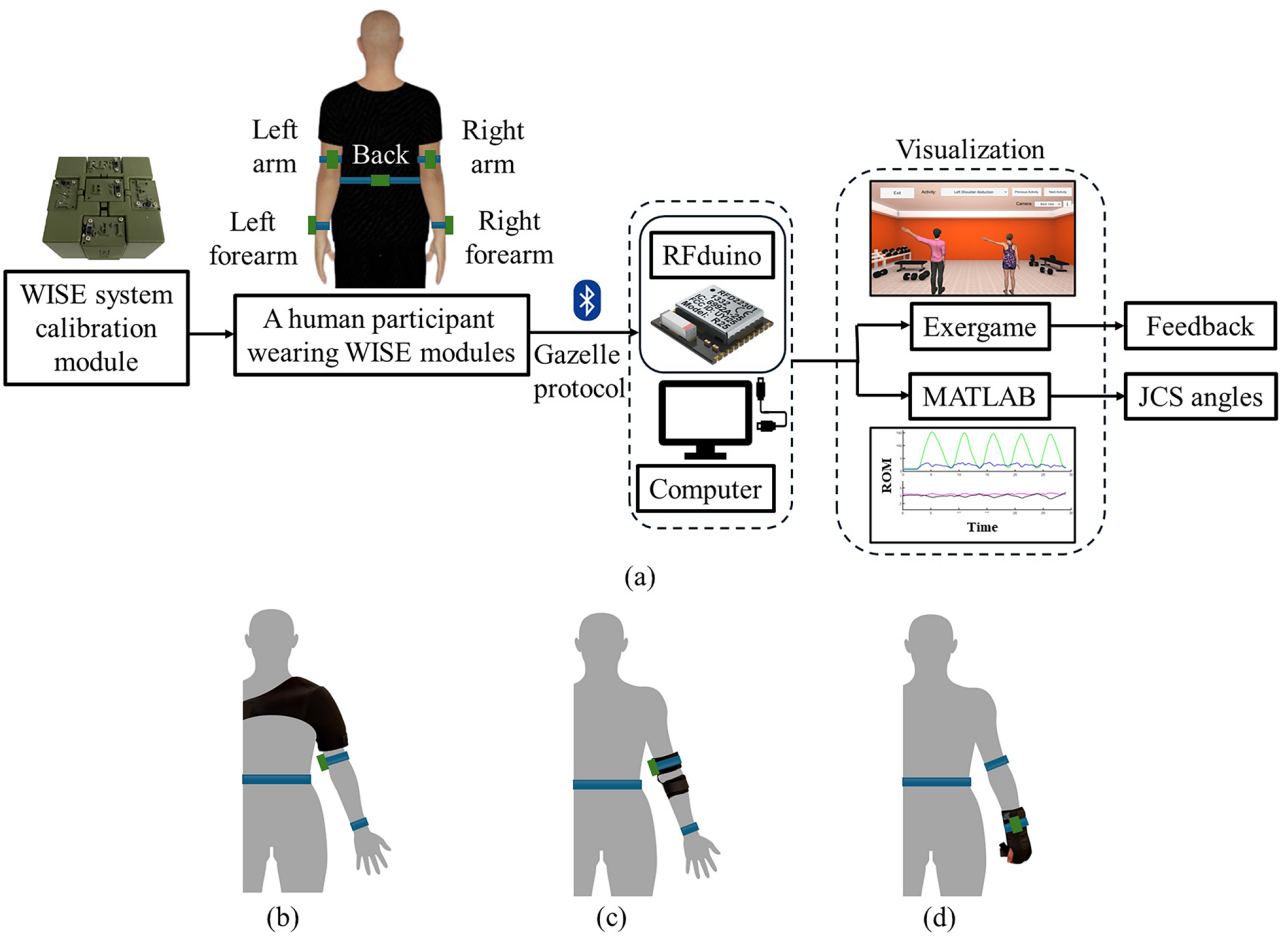
**a** An illustration of the hardware and software modules of the WISE system and a participant wearing the WISE modules and a participant wearing **b** shoulder, **c** elbow, and **d** forearm braces

The WISE system was used for ROM assessment in upper limb joints [24]. The hardware and software modules of the WISE system are shown in Fig. 1a. The system consists of five sensor modules, one worn on the back and two worn on each arm, attached using Velcro straps. It is used to measure the ROM of three rotational DOFs at the shoulder and one rotational DOF each at the elbow and forearm for each arm.

Every sensor module consists of an RFduino microcontroller that obtains from an on-board inertial measurement unit (IMU) the quaternion orientation measurement and wirelessly transmits it to a host RFduino microcontroller that is tethered to a computer. The quaternion measurements obtained from the IMU sensors are transformed by the software module of the WISE system to provide the angular joint excursions in the joint coordinate system (JCS) [28]. Note that the usability of the WISE system was validated for both left and right arm movements in Ref. [22]. Using the WISE hardware and software system, we obtained the joint trajectories for movements at the shoulder (flexion–extension, abduction–adduction, and internal–external rotation), elbow (flexion–extension), and forearm (pronation-supination). The joint movements were visualized in real-time on a Unity-based exergame platform [29] and graphed in MATLAB. To simulate restrictions at the shoulder, elbow, and forearm, we used a shoulder brace (Vive Health, Naples, Florida), an elbow brace (Drnaiety, Henan, China), and a forearm brace (FLA Orthopedics, Charlotte, North Carolina), respectively, as shown in Fig. 1b–d.

### Protocol

**Fig. 2:**
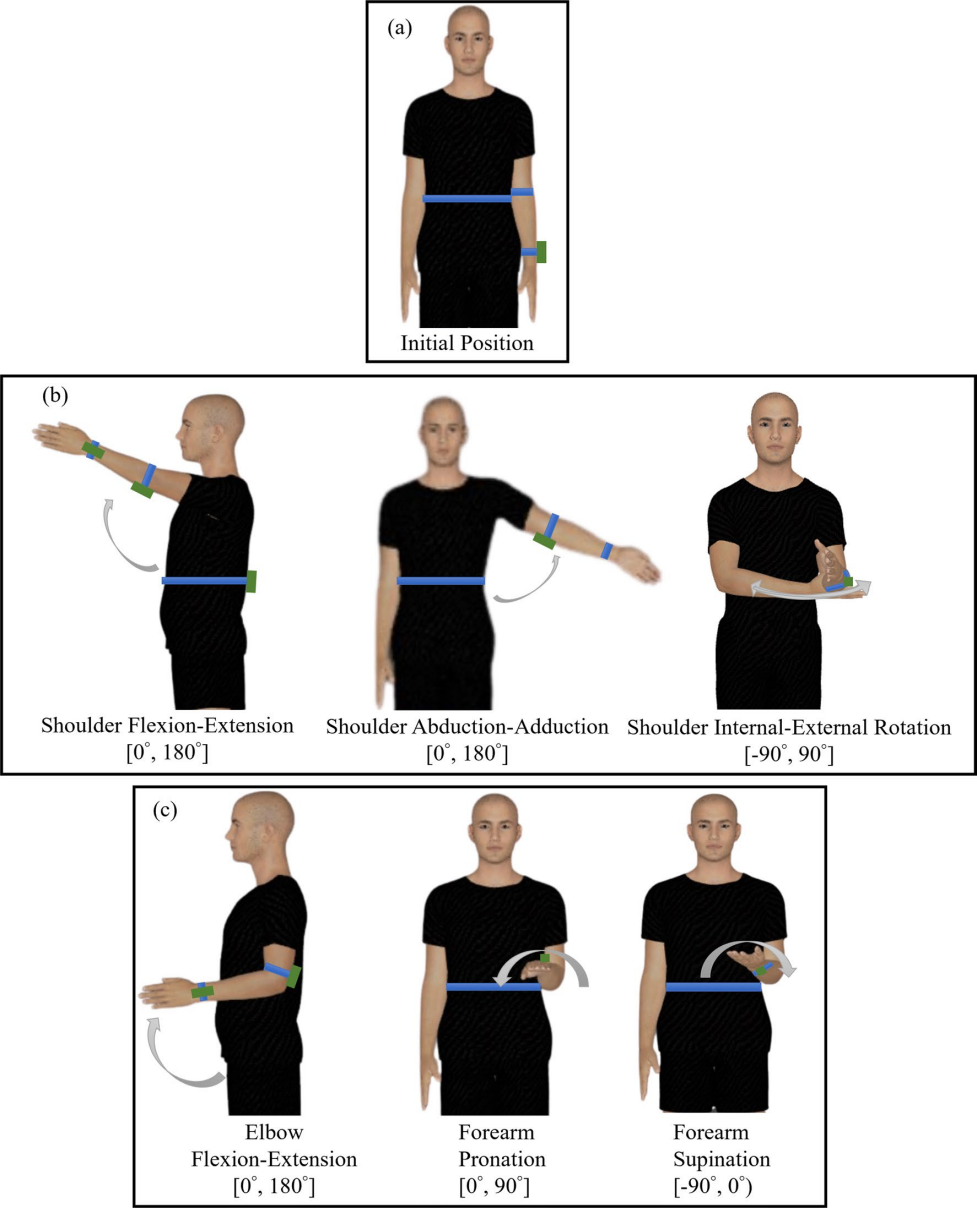
**a** Initial position and instructed movements for **b** shoulder and **c** elbow and forearm

Prior to being fitted with the WISE modules or the restriction braces, each participant was instructed on the sequence of movements to be performed through a video demonstration. Next, the participants were asked to practice each movement to ensure understanding and consistency in the start and end positions, which facilitated data interpretation. First, each participant performed a set of five trials of three shoulder movements (flexion–extension, abduction–adduction, and internal–external rotation), one elbow movement (flexion–extension), and one forearm movement (pronation-supination) with their unrestricted left arm while wearing the WISE sensors as shown in Fig. 2. Since this study was not focused on assessing the differences in ROM of instructed joint movements between left and right arms, the participants were asked to perform the instructed movements only with their left arm. While the selection of the non-dominant arm in the study may be deemed arbitrary, it nonetheless ensured consistency across participants, and avoided the potential effect of arm dominance on the ROM. Next, the participants were instructed to repeat the five movements for five trials with the restriction braces. Specifically, they performed the five movements under the following four experimental conditions: (1) unrestricted, (2) restricted shoulder, (3) restricted elbow, and (4) restricted forearm. The participants were provided rest breaks between each set of movements to avoid fatigue.

### Data processing and statistical analysis

For each condition, the joint excursion data corresponding to each of the five movements were collected and stored using MATLAB and analyzed offline using Rstudio [30]. For every participant, we computed the ROM for each trial (*i* = 1, …,5) of each instructed joint movement (*j* = 1, …,5) in each experimental condition (*k* = 1, …,4). Note that *j* = 1, 2, and 3 correspond to the shoulder flexion–extension, abduction–adduction, and internal–external rotation movements, respectively, while *j* = 4 and 5 correspond to the elbow flexion–extension and forearm pronation-supination movements, respectively.

For the movements of shoulder flexion–extension, shoulder abduction–adduction, and elbow flexion–extension, for each participant, across five trials of each movement, the *mean* of peak ROM for an instructed joint was calculated as follows

1 $$M_{jk} = \frac{{\sum_{i = 1}^5 p_i }}{5},$$

where ${p}_{i}$ is the peak ROM in the *i*th trial of movement, *i* = 1, …,5, and ${M}_{jk}$ is the mean of peak ROM of the instructed joint for the *j*th movement, *j* = 1, 2, 4, corresponding to the *k*th experimental condition, *k* = 1, …,4. Next, for the movements of shoulder internal–external rotation and forearm pronation-supination, the ROM can take both positive and negative values (shoulder internal rotation [0°,90°], shoulder external [− 90°,0°), forearm pronation [0°,90°], forearm supination [− 90°,0°)). Thus, for these movements, across five trials of each movement performed by each participant, the *mean* span of ROM of the instructed joint, for each experimental condition, was calculated using

2 $$S_{jk} = \frac{{\sum_{i = 1}^5 \left( {p_i - t_i } \right)}}{5},$$

where ${t}_{i}$ is the trough of respective joint ROM for the ${i}^{\text{th}}$ trial of the movement and ${S}_{jk}$ is the mean span of the ROM of the instructed joint for the ${j}^{\text{th}}$ movement, *j* = 3, 5, performed in the *k*th experimental condition.

Using the mean ROM data for each of the 20 participants, calculated from Eqs. (1) and (2), the *average* ROM of 20 participants and the corresponding standard deviation for each movement in every experimental condition were calculated. The ROM data were first examined for normality and homogeneity of variance assumptions using the Shapiro–Wilk test [31] and Levene’s test [32], respectively. The results indicated that the ROM datasets largely satisfied the assumptions to an acceptable level with some exceptions. Since analysis of variance (ANOVA) is known to be minimally sensitive to violations in normality and homogeneity assumptions [33–35], we used the one-way ANOVA test (*F* statistic) for each instructed movement to examine if the average ROM was statistically significantly different in one or more experimental conditions. To further identify if a restriction caused a statistically significant effect on the average ROM for an instructed joint, we performed post hoc pairwise comparisons using the Tukey tests (*q* statistic) [33]. Finally, to quantify the effect of a restriction on an instructed joint movement, the average percentage change in ROM of the instructed joint movement was calculated using

3 $$\Delta {\text{ROM}}\% = \frac{{\left( {{\text{ROM}}_{{\text{Unrestricted}}} - {\text{ROM}}_{{\text{Restricted}}} } \right)}}{{{\text{ROM}}_{{\text{Unrestricted}}} }} \times 100,$$

where ${\text{ROM}}_{{\text{Unrestricted}}}$ is the average ROM of all participants for the instructed movement in the unrestricted condition and ${\text{ROM}}_{{\text{Restricted}}}$ is the average ROM of all participants for the instructed movement in the restricted condition.

## Results

The instructed joint average ROM (i.e., µ) and corresponding standard deviation (i.e., σ) are presented in Table 1 for all four experimental conditions. We found that the data in both unrestricted and restricted joint conditions for all the instructed movements were within the normative range of shoulder [36, 37], elbow [38], and forearm [38] movements.

**Table 1:** ROM in degrees (µ (σ)) for instructed movements under unrestricted and simulated restriction conditions^‡^

Instructed movement		Shoulder F/E	Shoulder A/A	Shoulder I/E	Elbow F/E	Forearm P/S
Simulated condition	Unrestricted	159.24 (7.24)	159.03 (9.44)	90.81 (18.14)	139.91 (10.47)	149.11 (17.53)
	Restricted shoulder	111.31 (20.84)	98.57 (25.17)	49.24 (14.88)	109.23 (19.72)	139.55 (19.99)
	Restricted elbow	130.94 (18.17)	127.53 (19.30)	82.97 (21.01)	57.79 (13.13)	128.18 (17.23)
	Restricted forearm	149.64 (13.12)	148.12 (16.72)	75.63 (16.61)	125.90 (13.84)	101.46 (20.56)

^‡^Flexion–extension (F/E), abduction–adduction (A/A), internal–external rotation (I/E), and pronation-supination (P/S)

As expected and seen from Table 1, restricting the shoulder, elbow, or forearm reduced the average ROM for the corresponding instructed movements. Interestingly, the restrictions additionally reduced the average ROM for instructed movements of non-restricted joints. That is, we observed that restricting an instructed or a non-instructed joint reduced the average ROM for the instructed joint movements. The average percentage reduction in the ROM for each restricted *versus* unrestricted condition is computed and provided in Table 2a.

**Table 2:** For each instructed movement: (a) average percentage reduction in ROM and (b) results of post hoc Tukey tests (*q* values, *p* < 0.05 denoted using ^*^)

Instructed movement		Shoulder F/E	Shoulder A/A	Shoulder I/E	Elbow F/E	Forearm P/S
(a)						
Simulated disability	Restricted shoulder	30.09	38.01	45.77	21.93	6.41
	Restricted elbow	17.77	19.80	8.64	58.69	14.04
	Restricted forearm	6.03	6.86	16.71	10.01	31.96
(b)						
Comparison conditions	Unrestricted *versus* restricted shoulder	13.63^*^	14.58^*^	10.44^*^	9.34^*^	2.26
	Unrestricted *versus *restricted elbow	8.05^*^	7.60^*^	1.97	25.01^*^	4.96^*^
	Unrestricted *versus*restricted forearm	2.73	2.63	3.81^*^	4.27^*^	11.28^*^

Given the inter-subject variability in the mean ROM (indicated by standard deviation in Table 1), we used inferential statistical analysis to examine whether restricting a non-instructed joint had a statistically significant impact on the average ROM of the instructed joint movement. ANOVA test results revealed that at least one or more joint restrictions had a statistically significant effect on the average ROM for each instructed movement (shoulder F/E: *F* = 36.37^*^, shoulder A/A: *F* = 41.14^*^, shoulder I/E: *F* = 20.55^*^, elbow F/E:* F* = 119.36^*^, forearm P/S: *F* = 23.81^*^, at *p* < 0.05). Next, pairwise comparisons using Tukey test were performed to contrast the average ROM for each instructed movement between the unrestricted and restricted conditions (see Table 2b). Using Table 2, the following remarks are drawn. Since it is obvious that restriction at a particular joint will reduce the ROM at that joint, we highlight the effects of restrictions at non-instructed joints on the ROM of instructed joint movements. Note that restricting the shoulder led to a statistically significant reduction in the average ROM for elbow flexion–extension but not for forearm pronation-supination. Next, restricting the elbow reduced the average ROM for shoulder joint movements with statistically significant results for flexion–extension and abduction–adduction, but not for internal–external rotation, as well as for forearm pronation-supination. However, note that the percentage reduction in the average ROM was smaller for forearm pronation-supination than for the two shoulder movements. Finally, forearm restriction caused a statistically significant reduction in the average ROM for shoulder internal–external rotation and elbow flexion–extension. Thus, restricting the movement of specific non-instructed joints was seen to restrict the movement of an instructed joint.

**Fig. 3:**
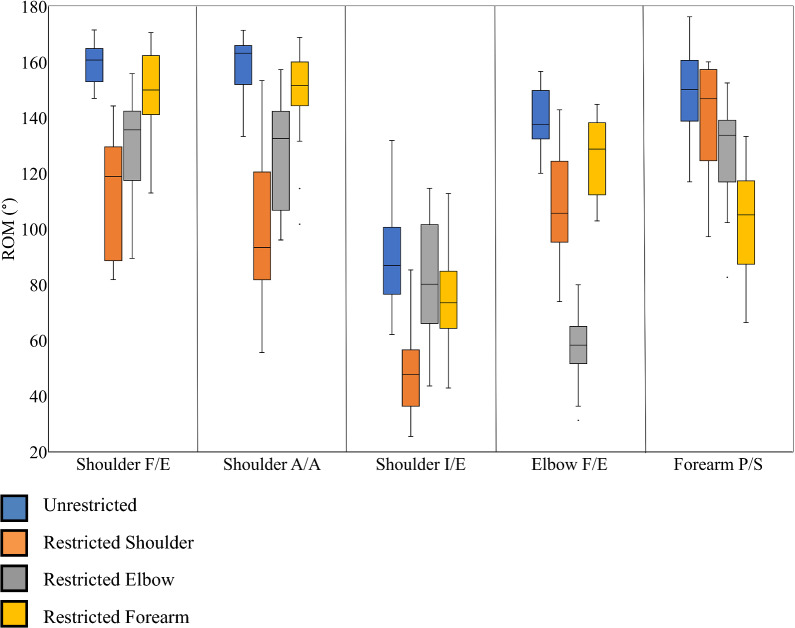
Distribution of ROM across unrestricted and restricted conditions for shoulder, elbow, and forearm

The box-and-whisker plots [33, 39] of Fig. 3 demonstrate the effects of specific joint interactions on the ROM of instructed joints. Shoulder flexion–extension and abduction–adduction are affected by the elbow restriction, as evidenced from the non-overlapping interquartile range of ROM, but not by the forearm restriction. In contrast, shoulder internal–external rotation is affected by the forearm restriction but not by the elbow restriction. Next, elbow flexion–extension is affected by restrictions at the shoulder and forearm. Finally, forearm pronation-supination is least affected by restriction at the shoulder and moderately affected by the elbow restriction. Furthermore, for all instructed joint movements, the ROM data under the three restriction conditions showed a greater spread than the ROM data under the unrestricted condition, suggesting greater inter-subject variability in response to restrictions. This greater inter-subject variability in restricted *versus* unrestricted conditions could be due to differing responses of participants to restrictions imposed on the joints. Overall, we found only four outliers in the box-and-whisker plot representation of ROM datasets in Fig. 3, particularly: two in shoulder abduction–adduction performed with forearm restriction as well as one in elbow flexion–extension and one in forearm pronation-supination performed with elbow restriction. These outlier data points fell below the lower whisker of the corresponding box plot, indicating that the imposed restriction resulted in a higher loss of ROM for these participants compared to others for these specific movements.

## Discussion

In this work, we sought to examine the effect of joint interaction forces arising from the non-instructed joints on the ROM of instructed joint movements using the disability simulation method. We found that restrictions at the non-instructed joints significantly reduced the ROM of several instructed movements, suggesting that the joint interaction forces from non-instructed joints aid ROM at the instructed joints. Specifically, shoulder restriction significantly reduced ROM for instructed elbow flexion–extension but not for instructed forearm pronation-supination. In addition, elbow restriction significantly reduced ROM for instructed shoulder flexion–extension, instructed shoulder abduction–adduction, and instructed forearm pronation-supination, but not for instructed shoulder internal–external rotation. Finally, forearm restriction significantly reduced ROM for instructed shoulder internal–external rotation and instructed elbow flexion–extension. These results are discussed below.

### Advantages of simulating disability in examining the role of joint interaction forces

Biomechanically, the arm is composed of multiple linked joints. During natural movements, forces generated by muscles and tendons at an instructed joint are influenced by mechanical forces such as gravity as well as forces produced by muscles and tendons at other joints, which interact to increase or decrease the movement at the instructed joint [12–15, 40–42]. In healthy individuals, in response to these joint interaction forces, the central control signals to muscles are adjusted [40]. Control of these joint interaction forces necessitates the activation of certain muscles across many varied movements and forms the basis of muscle synergies [43]. However, in individuals with movement deficits, which may result from sensory impairment such as loss of proprioception [13, 14], motor impairment such as ataxia [12], muscle weakness as in muscular dystrophy [44], or a combination of lack of central coordination, weakness, and spasticity such as in stroke [13, 45–47], impaired ability to control the joint interaction forces may contribute to compensatory movements that may exacerbate the movement deficit. Investigating the effects of disease-induced joint restrictions on specific instructed movements using real-world 3D ROM data analysis, for example using the WISE system, may reveal the person-specific patterns of deterioration (e.g., percentage reduction) that could then be used to tailor individualized treatments.

A disability simulation study is the ideal way to test the role of joint restrictions on joint interaction forces for at least three reasons. First, unrestricted movements cannot be tested in patient populations, hence there is no control. Even the “unaffected” arm in individuals with stroke shows evidence of impairment [48]. Second, patient populations may experience simultaneous restrictions at multiple joints, making it difficult to understand the effects of restriction at one joint at a time in the same individual [5]. Third, as we have seen large standard deviations in the restricted conditions, individuals may be able to overcome the resistance from the restrictions to varying degrees, producing large inter-individual variability. In fact, in patient populations where the impairment often includes weakness along with resistance to joint motion, the inter-individual variability is further increased, making it difficult to draw clear conclusions [49].

### Role of joint interactions in the ROM of upper limb joint movements

It has been shown that the interaction between two adjacent joints plays a key role in many functional multi-joint activities such as reaching, drawing, and throwing [41, 42, 50]. Our results also show that restrictions at adjacent joints have a larger effect in reducing ROM at the instructed joint, especially when the restriction is placed on the shoulder or elbow. For example, our data show that the shoulder restriction led to ~ 22% reduction for instructed elbow flexion–extension. One explanation for this may be the anatomy and action of muscles that cross the two joints. Specifically, the long head of the biceps brachii muscle [51], which extends from the scapula bone in the shoulder girdle to the radial bone in the forearm, controls flexion at the elbow. Thus, the shoulder brace may impede the action of biceps brachii, although we did not explicitly measure its activity.

Notably, restriction at the elbow led to a slightly greater reduction in shoulder abduction–adduction than it did for shoulder flexion–extension. Anatomically the pectoralis major muscle, which is involved in both shoulder adduction and flexion, sends a large myofascial expansion to the anterior region of the fascia over the biceps muscle, and the biceps brachii sends a large myofascial expansion to the medial aspect of the forearm, the lacertus fibrosus [52–54]; restriction of these myofascial expansions with an elbow brace can thus limit shoulder flexion–extension and abduction–adduction, as well as forearm pronation-supination, as evidenced in Table 2a. Individuals with stroke tend to show a flexor synergy pattern that mimics the pattern of movement reduction seen with an elbow brace, which may be related to stiffness of the myofascial expansions around the elbow [55]. Indeed, reducing the stiffness across the pectoral and upper arm muscles in individuals with a flexor synergy pattern after cerebral injury has been shown to increase the movement at the shoulder, elbow, and forearm [56].

Interestingly, forearm restriction significantly reduced ROM for shoulder internal–external rotation even though the forearm brace did not directly limit the function of muscles involved in shoulder internal–external rotation. Mechanical interactions between limb segments can explain joint interaction forces that produce motions without muscle contraction [42]. Forearm rotation is produced due to rotation at the proximal and distal radioulnar joint as well as due to rotation of the whole upper limb at the shoulder [57]. The forearm and upper arm are connected by their respective ulna bone and humerus bone at the humeroulnar joint [57]. The position of the ulna may thus control the position and orientation of the humerus at the glenohumeral and scapulothoracic joints [57]. Thus, mechanical restriction of the forearm (ulna bone) leading to restriction of the humeroulnar joint may cause a reduction in shoulder internal–external rotation.

### Validity of using the WISE system for real-world ROM assessments

We used the WISE system [24] to obtain real-world 3D ROM data of instructed movements at the shoulder, elbow, and forearm in unrestricted and simulated disability conditions. In the unrestricted condition, the ROM results for all joint movements for all participants were consistent with those published in previous literature [36–38]. This validates the WISE system as a tool to reliably measure 3D ROM across multiple upper limb joints selectively in a real-world setting. Additionally, we found that in the unrestricted condition, the ROM measurements had smaller standard deviations, which is suggestive of low inter-subject variability in these healthy individuals. Next, as expected, we found that restricting an instructed joint significantly decreased the ROM at that joint while increasing the inter-subject variability, perhaps due to the differing abilities of individuals to overcome the joint restrictions. It is well-documented that patient populations demonstrate higher inter-subject variability; for example, hemiparetic patients show higher variability in the ROM of instructed flexion–extension movements of the shoulder, elbow, and wrist compared with healthy controls [58].

Current rehabilitative interventions do not benefit from the information accessible through kinematic measurements of joint ROM and evaluation of compensatory patterns and joint interactions. Overcoming this drawback necessitates greater availability of easy-to-use, user-friendly, kinematic measurement tools that provide real-time results to be acted upon by clinicians. These kinematic measurements must be clinically acceptable, facilitate rather than encumber care, have adequate resolution to provide relevant information that can guide treatment, and reduce barriers to accessing care. Several attempts are currently underway to make this feasible through IMU-based, visual marker-based [59], and marker-less MOCAP [60, 61] solutions. In this vein, the work of this paper shows the utility of the WISE system for real-world 3D measurements of shoulder, elbow, and forearm movements under various simulated disability conditions and uncovers the patterns of joint responses that have not been revealed previously.

### Limitations

This work has several shortcomings that should be considered when examining its findings for generalization. First, even as our ROM measurements were within the normative range [36–38], these measurements cannot be compared with measurements in prior works [17, 59–62] due to different techniques used for joint angle estimation and ROM measurement. Second, the variability in the data may arise partly due to sources of errors from the measurement system (e.g., sensor drift and calibration error) or limitations in simulating disability rather than actual participant variability. Third, the current study did not measure electromyographic activity to compare muscle activation patterns across experimental conditions. Participants may have differences in muscle activation to overcome the resistance from the joint restrictions, explaining the variability in joint ROM. It has been shown that muscle activation patterns when combined with movement better explain the inter-individual variability, particularly in muscle synergy patterns, after a stroke [63]. Fourth, the small sample size and narrow age range of participants limits generalization of the results. Fifth, this study did not consider the effect of arm dominance on ROM data, as all the participants performed movements with their left non-dominant arm. Although prior research [37, 64] found arm dominance to have a significant effect on shoulder ROM, it was deemed to be likely lacking in clinical relevance [37]. Sixth, this study did not consider the effect of gender on ROM data, although prior research has found a significant difference in shoulder joint ROM between genders [37]. Additionally, Ref. [17] has found a significant difference in the ROM for forearm pronation between genders but no such difference for other upper limb movements. Finally, [18] has reported significant gender differences in upper limb joint angles involved during the task of eating.

## Conclusion

This study examined the impact of the shoulder, elbow, and forearm restrictions on the ROM for the three instructed movements of shoulder (flexion–extension, abduction–adduction, and internal–external rotation) and one instructed movement each of elbow (flexion–extension) and forearm (pronation-supination). To the best of our knowledge, a comprehensive investigation of the role of joint interactions on the ROM of upper limb joint movement using simulated disability has not been performed in prior research. The findings elucidate the role of muscular, myofascial, and mechanical interactions across non-instructed joints in reducing the movement at instructed joints. Furthermore, this study validates the use of the WISE system for real-world ROM measurements that could be helpful to assess the role of impairments and inform rehabilitation in patient populations. Future work should consider: (i) simultaneously capturing ROM and muscle activation data to provide a better understanding of muscle synergies and joint interaction forces in unrestricted *versus* restricted conditions; (ii) assessing the differences in ROM and motor control between dominant and non-dominant limbs in restricted conditions; and (iii) using a larger sample size to increase the power of statistical analysis and enhance the reliability of results for clinical applications.

## Data Availability

The datasets generated and analyzed during this study are available from the corresponding author on reasonable request.

## Abbreviations

3D: Three dimensional
A/A: Abduction-adduction
ADLs: Activities of daily living
ANOVA: Analysis of variance
DOF: Degree of freedom
F/E: Flexion-extension
IMU: Inertial measurement unit
I/E: Internal-external rotation
JCS: Joint coordinate system
MOCAP: Motion capture
P/S: Pronation-supination
ROM: Range of motion
WISE: Wearable inertial sensors for exergames
